# A Community Health Worker–Led Community–Clinical Linkage Model to Address Emotional Well-Being Outcomes Among Latino/a People on the US–Mexico Border

**DOI:** 10.5888/pcd18.210080

**Published:** 2021-08-05

**Authors:** Abby M. Lohr, Kevin Doubleday, Maia Ingram, Ada M. Wilkinson-Lee, Kiera Coulter, Karl Krupp, Cynthia Espinoza, Floribella Redondo-Martinez, Cassalyn David, Scott C. Carvajal

**Affiliations:** 1 Arizona Prevention Research Center at the Mel and Enid Zuckerman College of Public Health, University of Arizona, Tucson, Arizona; 2 Mexican American Studies Department, College of Social and Behavioral Sciences, University of Arizona, Tucson, Arizona; 3 Yuma County Health District, Yuma, Arizona; 4 Arizona Community Health Workers Association, Yuma, Arizona; 5 Mariposa Community Health Center, Nogales, Arizona

## Abstract

**Introduction:**

Compared with their non-Hispanic White counterparts, Latino/a people have limited access to health resources that might improve their emotional well-being. Interventions that prioritize the Latino/a population, address social determinants of health, and decrease health disparities are needed. The objective of this study was to describe a community–clinical linkage intervention led by community health workers (CHWs) in 3 Latino/a populations along the US–Mexico border.

**Methods:**

Researchers at the Arizona Prevention Research Center conducted the Linking Individual Needs to Community and Clinical Services (LINKS) study during 2017–2018. Clinic-based CHWs referred participants to community-based CHWs who met with participants monthly for 6 months to assess participant needs, provide support for emotional well-being, and link them to resources. Two community-based CHWs collaborated to maximize participant care; they also administered an emotional well-being questionnaire at baseline and at 3-month and 6-month follow-up. We estimated changes in emotional well-being outcomes.

**Results:**

Scores for social support, perceived hopefulness, and quality-of-life measures among 189 LINKS participants increased significantly during the study period, especially among men and participants with low baseline scores. For each of the 3 outcomes, the standardized change was approximately 0.28 per 3 months of intervention, a decrease of more than half an SD (0.56) during 6 months of follow-up.

**Conclusion:**

A CHW-led community–clinical linkage intervention can result in positive emotional well-being outcomes. We encourage policy makers, funders, and public health practitioners to further investigate such interventions as a solution to reduce disparities in emotional well-being.

**Summary**

**What is already known on this topic**

Community health workers (CHWs) effectively disseminate behavioral interventions that improve participant health outcomes in historically disenfranchised communities. More research is needed to examine how CHWs can create successful linkages to further improve emotional well-being outcomes and enhance social and cultural resilience factors consistent with Latino/a health advantages.

**What is added by this report?**

We described an example of a CHW-led community–clinical linkage where CHWs collaborated to improve emotional well-being outcomes among participants.

**What are the implications for public health practice?**

As clinic and community organizations are increasingly integrating CHWs into their work, it is crucial that evidence-based examples of CHW-led community–clinical linkages are available to support these efforts.

## Introduction

Social determinants that negatively affect the health of Mexican Americans are magnified along the US–Mexico border, where several factors coalesce to make the region a challenging place to live. Border residents are twice as likely than the population of any individual US state to live in poverty, attend fewer years of school, and experience higher rates of unemployment (1). These social determinants translate into social and economic contexts that influence health. People of Mexican origin in border communities with high levels of poverty and chronic disease face additional barriers ― beyond cost and lack of insurance ― to accessing health care. Economic stress contributes to denial of illness and delay in seeking health care until advanced illness, which is then exacerbated by poor interpersonal interactions with health care providers (2). One study found that nearly two-thirds of adult Mexican Americans along the border had diabetes or prediabetes, and that half of adults with diabetes were undiagnosed. Even people diagnosed with diabetes struggled to access care (3). Financial barriers and tensions related to immigration status may lead to stress, depression, and/or anxiety among border residents who have few economic resources to care for their health (4,5).

Although the terms *mental health* and *emotional well-being* are often used synonymously, our study team used the construct of emotional well-being. Emotional well-being has been defined as the perception among people that “their lives are going well,” including “the quality of their relationships, their positive emotions and resilience, realization of their potential, or their overall satisfaction with life — ie, their ‘well-being’” (6,7). Emotional well-being stems from a broad, comprehensive view of health that includes positive outcomes and traditionally reviewed indicators such as quality of life.

One solution to reducing disparities in social determinants of health and emotional well-being among Latino/a people may be to use community health worker (CHW)-led community–clinical linkages (8). CHWs are frontline public health workers with a special connection to their community (9). We define community–clinical linkages as connections between community-based and clinical services to improve patient access to resources (10). CHWs have successfully delivered interventions on health education (11), preventive health screenings (12), behavior change (13), and chronic disease prevention and management (14). Although examples exist of CHW-led community–clinical linkages in the literature, more research is needed to examine how CHWs create linkages that improve emotional well-being outcomes (8).

The objective of this study was to describe a CHW-led community–clinical linkage intervention that prioritized a Latino/a population along the US–Mexico border (8). Linking Individual Needs to Community and Clinical Services (LINKS) was a 3-year, prospective matched observational study conducted by the Arizona Prevention Research Center. This project, guided by community-based participatory research principles, examined the impact of CHW-led community–clinical linkages on the US-Mexico border to address chronic disease management and prevention as well as emotional well-being. Here we present our analysis of data on emotional well-being outcomes among LINKS participants. 

## Methods

LINKS took place in 3 Arizona border counties: Pima, Yuma, and Santa Cruz. We collected data from June 2017 through September 2018. Community partners from 3 federally qualified health centers and 2 county health departments participated in the study. In Pima and Yuma counties, we created a linkage between a clinic-based CHW and a community-based CHW who was employed by the local county health department. Santa Cruz County has a small population and as a result, the local federally qualified health center also serves as the county health department. Therefore, in Santa Cruz County, we formed a community–clinical linkage across clinic departments. Details on LINKS methodology are available elsewhere (6). Our research was approved by the University of Arizona Institutional Review Board.

Staff members from the Arizona Community Health Workers Association (AzCHOW) trained the CHWs by using the Behavioral Health Training for Community Health Workers in Primary Care (15), Sonrisa (a curriculum on addressing comorbid depression and diabetes among Latinos) (16), and Mental Health First Aid (17). Additionally, AzCHOW staff members assessed the skills of each CHW and held monthly group trainings throughout the intervention in which CHWs could learn from their peers, build on their strengths, and gain new knowledge and abilities.

The 6-month, one-on-one, client-driven LINKS intervention proceeded as follows. First, the clinic-based CHW recruited potential participants who met the inclusion criteria (consenting adults [aged ≥18 y] with diagnosed chronic disease or pre–chronic disease) and referred them to the community-based CHW. The community-based CHW also recruited people who met the inclusion criteria from community events such as health fairs. Once recruited, the community-based CHW then built rapport and trust, provided emotional support (eg, taught relaxation techniques and stress management skills), and helped the participant navigate any needed services during monthly meetings (or more often as needed). As the community-based CHW built trust during each of the 6 follow-up visits, they created a supportive and reliable relationship for participants. Therefore, the core elements of the intervention were not focused on a disease state but were tailored to each participant’s needs and geared toward improving overall quality of life.

While forming a relationship and linking participants to resources, the LINKS community-based CHWs also collected data. Using an iPad, they administered the emotional well-being questionnaire at baseline and at 3-month and 6-month follow-up. They entered participant responses into a secure Research Electronic Data Capture (REDCap) database (18). Instead of direct translation, the Arizona Prevention Research Center team translated survey questions by using functional adequacy of the translation (coming to consensus about the functional meaning of the question). This approach is an alternative to back-translation and can result in high-quality translations in cross-cultural research (19).

### Data collection instruments

To collect data on emotional well-being, we used 3 instruments. These instruments were adapted from 1) the Social Support Inventory (20), 2) the State Hope Scale (21), and 3) the Short-Form 8 (SF-8), hereinafter referred to as quality of life (22). Each participant could have a maximum of 3 records for each scale: one each at baseline, 3-month follow-up, and 6-month follow-up.

**Social Support Inventory.** This instrument used a 7-item scale to measure social support. Responses for items 1 through 6 were scaled from 1 (none of the time) to 5 (all the time). For example, item 1 was “Is there someone available to you whom you can count on to listen to you when you need to talk?” Item 7 asked, “Are you currently married or living with a partner?” and was recorded as 2 (no) and 4 (yes). Social Support Inventory scores were calculated at each follow-up by summing responses from the 7 items; scores ranged from 8 to 34 (α = 0.81 at baseline), with higher scores indicating greater perceived levels of social support.

**State Hope Scale.** This instrument used a 6-item scale to measure ongoing, goal-directed thinking. The aim is to assess whether a respondent feels they can improve their life and see a path to achieving this improvement. Responses for all items were scaled from 1 (none of the time) to 5 (all the time). As an example, item 1 was “If I should find myself in a jam, I could think of many ways to get out of it.” State Hope Scale scores were calculated at each follow-up by summing responses from the 6 items. State Hope Scale scores ranged from 6 to 30 (α = 0.87 at baseline), with higher scores indicating greater levels of hopefulness.

**Quality of life.** To examine the culture and language of LINKS participants (19), 8 items from the SF-8 quality-of-life instrument were adapted and translated to our local context. Responses for items 1 and 4 were on a scale from 1 to 6 and the remaining items from 1 to 5. For example, item 1 was “Overall, how would you rate your health during the past 4 weeks?” and was scored as 1 (excellent) to 6 (very poor). Item 2 was “During the past 4 weeks, how much did physical health problems limit your physical activities (such as walking or climbing stairs)?” and was scored as 1 (could not do physical activities) to 5 (not at all). Unlike the standard SF-8 in English, our adapted instrument did not have existing normative values. Thus, we represented quality of life by summing responses from the 8 items, which were internally consistent (α = 0.88 at baseline). These scores ranged from 8 to 42, with higher scores indicating better perceived quality of life. 

### Statistical analysis

Baseline covariate summaries for the 3 clinical groups, along with the entire study sample, were reported as mean (SD) for continuous measures and frequency (percentage) for categorical measures. We used analysis of variance for continuous measures and the Fisher exact test for categorical measures to compare clinical groups at baseline. Linear mixed-effects models were fit separately for each scale. The response was the total score from the given scale. We included mixed effects for time point (ie, 0, 3, 6 months), age, sex (male/female), years of education (≤12, 13–16, and >16), and interactions of age and sex with time point. To account for the fact that observations for each participant were likely to be correlated across follow-up, we included random effects for participant (ie, participant-level random intercept) and participant trajectory over time (ie, patient-level random slope). We considered 2 cohorts: a full cohort, comprising all participants with available data, and a reduced cohort, comprising participants with baseline scale scores in the lower 3 quartiles. We conducted the primary analysis on the full cohort and exploratory analyses on the reduced cohort, aiming to estimate the intervention effect among LINKS participants with lower scores at baseline (ie, participants who had a chance for measured improvement). All analyses were conducted using statistical software R version 3.3.3 (R Foundation).

## Results

Of the 189 LINKS participants, 148 (78.3%) completed the 3-month follow-up survey (21.7% attrition) and 172 (91.0%) completed the 6-month follow-up survey (9.0% attrition). A total of 146 (77.2%) LINKS participants completed all 3 follow-up surveys, and 174 (92.0%) completed at least 2 of 3 follow-up surveys.

Most (85.7%) participants were women; the mean age was 56.6 (SD, 13.9) years (Table 1). One clinic had a study population that was considerably older than the others (Clinic 2 mean age, 67.7 [SD, 12.7] y; analysis of variance, *P* < .001). Across all clinics, the LINKS participants identified nearly exclusively as Latino/a (94.2%). Educational attainment was similar among the 3 clinics (*P* = .07); 72.5% of participants had 12 or fewer years, 18.7% had some college, and 7.1% attended graduate school. Baseline mean Social Support Inventory scores were similar across clinics (*P* = .60). Baseline mean State Hope Scale and quality of life scores were different across clinics (*P* < .001 for both instruments). Clinic 1 had higher mean (SD) State Hope Scale scores (25.0 [4.3]) than clinics 2 and 3 (21.8 [4.8]; 22.2 [6.3], respectively). Clinic 3 had lower mean (SD) quality of life scores (27.8 [7.8]) than clinics 1 and 2 (32.0 [6.0]; 32.4 [6.7], respectively).

**Table 1:** Baseline Characteristics of Participants in Linking Individual Needs to Community and Clinical Services (LINKS), a Community–Clinical Linkage Intervention in 3 Clinics Serving Primarily Latino/a Populations Along the US–Mexico Border, 2017-2018^a^

Characteristic	Clinic 1 (n = 93)	Clinic 2 (n = 26)	Clinic 3 (n = 70)	Total (n = 189)	*P* Value^b^
**Female sex, n (%)**	79 (84.9)	24 (92.3)	59 (84.3)	162 (85.7)	.66
**Age, mean (SD), y**	52.8 (12.7)	67.7 (12.7)	57.5 (13.8)	56.6 (13.9)	<.001
**Ethnicity, n (%)**					
Latino/a	91 (97.8)	26 (100.0)	62 (88.6)	179 (94.2)	.64
Non-Latino/a	2 (2.2)	0	8 (11.4)	11 (5.8)	
**Birth country, n (%)**					
Mexico	79 (84.9)	24 (92.3)	52 (74.3)	155 (82.0)	.27
United States	12 (12.9)	2 (7.7)	16 (22.9)	30 (15.9)	
Other	2 (2.2)	0	2 (2.9)	4 (2.1)	
**Years of education, n (%)**					
≤12	66 (71.7)	20 (76.9)	46 (71.9)	132 (72.5)	.07
13–16	22 (23.9)	3 (11.5)	9 (14.1)	34 (18.7)	
>16	3 (3.2)	1 (3.8)	9 (14.1)	13 (7.1)	
No response	1 (1.1)	2 (7.7)	0	3 (1.6)	
**Emotional well-being measures**					
Social Support Inventory, mean (SD)^c^	27.3 (5.5)	26.9 (7.3)	26.3 (6.5)	26.9 (6.2)	.60
State Hope Scale, mean (SD)^d^	25.0 (4.3)	21.8 (4.8)	22.2 (6.3)	23.5 (5.3)	<.001
Quality of Life, mean (SD)^e^^,^ f	32.0 (6.0)	32.4 (6.7)	27.8 (7.8)	30.5 (7.0)	<.001

a Mixed model incorporated clinic as a random effect (to account for similarities between people who attend the same clinic); however, the primary inferential statistics for this study reflect the overall effect across all data.

b Fisher exact test used for categorical variables and analysis of variance for continuous variables on available responses.

c A 7-item scale whose scores ranged from 8 to 34, with higher scores indicating greater perceived social support. In full cohort, n = 185 at baseline, n = 141 at 3-month follow-up, and n = 143 at 6-month follow-up. In reduced cohort, n =137 at baseline, n =106 at 3-month follow-up, and n = 108 at 6-month follow-up.

d LINKS effect is the estimated change in scale score for each of the 3-month follow-up periods. A total of 6 months of follow-up was completed for the LINKS study.

e A 6-item scale whose scores ranged from 6 to 30, with higher scores indicating hopefulness. In full cohort, n = 180 at baseline, n = 141 at 3-month follow-up, and n = 145 at 6-month follow-up. In reduced cohort, n =132 at baseline, n = 94 at 3-month follow-up, and n = 104 at 6-month follow-up.

f Eight quality-of-life items from the Short-Form 8 were adapted and translated to our local context; scores ranged from 8 to 43, with higher scores indicating better perceived quality of life. In full cohort, n = 183 at baseline, n = 144 at 3-month follow-up, and n = 145 at 6-month follow-up. In reduced cohort, n =131 at baseline, n = 99 at 3-month follow-up, and n = 99 at 6-month follow-up.

In the full cohort, the estimated change in the Social Support Inventory for each follow-up was +0.92 (95% CI, 0.48 to 1.35; *P* < .001), indicating an estimated increase in perceived social support of 1.84 units from baseline to 6-month follow-up, adjusting for sex, age, and education (Table 2). The overall effect of LINKS was larger in the reduced cohort (estimate = 1.68; 95% CI, 1.20 to 2.17; *P* < .001) than in the full cohort.

**Table 2:** Exploratory Analysis of Effect of Linking Individual Needs to Community and Clinical Services (LINKS) Intervention on Social Support, Hopefulness, and Quality of Life, Adjusting for Educational Attainment, Full Cohort and Reduced Cohort,^a^ a Community–Clinical Linkage Intervention in 3 Clinics Serving Primarily Latino/a Populations Along the US–Mexico Border, 2017-2018^a^

Parameter^a^	Full Cohort, Estimate (95% CI)	*P* Value^b^	Reduced Cohort, Estimate (95% CI)	*P* Value^b^
**Social Support Inventory^c^**				
LINKS (3-month change)^d^	0.92 (0.48 to 1.35)	<.001	1.68 (1.20 to 2.17)	<.001
Male sex	−0.09 (−2.65 to 2.46)	.94	−2.33 (−5.10 to 0.44)	.11
Age	−0.06 (−0.12 to 0.01)	.08	0.02 (−0.04 to 0.08)	.50
LINKS (3-month change) × male sex	0.45 (−0.79 to 1.71)	.48	0.65 (−1.06 to 2.35)	.46
LINKS (3-month change) × age	−0.01 (−0.04 to 0.02)	.34	−0.05 (−0.09 to −0.02)	.005
High school education	0.97 (−0.82 to 2.76)	.29	1.67 (−0.29 to 3.62)	.10
Some college education	1.74 (−0.26 to 3.74)	.09	2.28 (0.13 to 4.42)	.04
**State Hope Scale^e^**				
LINKS (3-month change)^d^	1.09 (0.76 to 1.42)	<.001	1.44 (1.06 to 1.82)	<.001
Male sex	2.42 (0.35 to 4.49)	.02	1.07 (−0.51 to 2.66)	.19
Age	0.02 (−0.03 to 0.07)	.40	0 (−0.04 to 0.04)	.99
LINKS (3-month change) × male sex	−0.42 (−1.38 to 0.53)	.39	−0.19 (−1.25 to 0.87)	.73
LINKS (3-month change) × age	−0.05 (−0.08 to −0.03)	<.001	−0.06 (−0.08 to −0.03)	<.001
High school education	2.03 (0.44 to 3.61)	.01	−0.08 (−1.34 to 1.19)	.91
Some college education	3.68 (1.89 to 5.48)	<.001	0.56 (−1.00 to 2.11)	.49
**Quality of Life^f^**				
LINKS (3-month change)^d^	1.04 (0.47 to 1.61)	<.001	0.92 (0.21 to 1.64)	.01
Male sex	0.26 (−2.60 to 3.12)	.86	−1.97 (−4.49 to 0.54)	.13
Age	0.02 (−0.05 to 0.09)	.56	0 (−0.06 to 0.06)	.91
LINKS (3-month change) × male sex	0.46 (−1.16 to 2.11)	.58	1.67 (−0.67 to 4.04)	.17
LINKS (3-month change) × age	−0.06 (−0.10 to −0.03)	.002	−0.06 (−0.11 to −0.01)	.02
High school education	1.95 (−0.13 to 4.03)	.07	1.48 (−0.31 to 3.27)	.11
Some college education	3.97 (1.63 to 6.30)	.001	2.60 (0.58 to 4.62)	.01

a Reduced cohort excludes participants with baseline scores in the top quartile (ie, people unlikely to be able to improve because they had high baseline scores).

b Calculated by using 2-tailed *t* test.

c A 7-item scale whose scores ranged from 8 to 34, with higher scores indicating greater perceived social support. In full cohort, n = 185 at baseline, n = 141 at 3-month follow-up, and n = 143 at 6-month follow-up. In reduced cohort, n =137 at baseline, n =106 at 3-month follow-up, and n = 108 at 6-month follow-up.

d LINKS effect is the estimated change in scale score for each of the 3-month follow-up periods. A total of 6 months of follow-up was completed for the LINKS study.

e A 6-item scale whose scores ranged from 6 to 30, with higher scores indicating greater hopefulness. In full cohort, n = 180 at baseline, n = 141 at 3-month follow-up, and n = 145 at 6-month follow-up. In reduced cohort, n =132 at baseline, n = 94 at 3-month follow-up, and n = 104 at 6-month follow-up.

f Eight quality-of-life items from the Short-Form 8 were adapted and translated to our local context; scores ranged from 8 to 43, with higher scores indicating better perceived quality of life. In full cohort, n = 183 at baseline, n = 144 at 3-month follow-up, and n = 145 at 6-month follow-up. In reduced cohort, n =131 at baseline, n = 99 at 3-month follow-up, and n = 99 at 6-month follow-up.

For the State Hope Scale, the estimated change for each follow-up was +1.09 (95% CI, 0.76–1.42; *P* < .001), indicating an estimated increase in perceived hopefulness of 2.18 units from baseline to 6-month follow-up, adjusting for sex, age, and education. The interaction of age across follow-up times was also significant (estimate = −0.05; 95% CI, −0.08 to −0.03; *P* < .001), indicating that for a fixed follow-up time, a 1-year increase in participant age was accompanied by a 0.05-unit decrease in State Hope Scale score. The increase in perceived hopefulness was 2.42 (95% CI, 0.35–4.49) units greater among men than among women (*P* = .02). In the reduced cohort, this sex effect was attenuated to 1.07 (95% CI, −0.51 to 2.66; *P* = .19). The overall effect of LINKS was larger in the reduced cohort than in the full cohort (estimate = 1.44; 95% CI, 1.06–1.82; *P* < .001).

The estimated change in quality of life for each follow-up was 1.04 (95% CI, 0.47–1.61; *P* < .001), indicating an estimated increase in perceived quality of life of 2.08 units from baseline to 6-month follow-up, adjusting for sex, age, and education. The estimate for the interaction of follow-up with age was significant (estimate = −0.06; 95% CI, −0.10 to −0.03; *P* = .002), indicating that for a fixed follow-up time, a 1-year increase in participant age was accompanied by a 0.06-unit decrease in self-report of quality of life. The overall effect of LINKS was slightly smaller in the reduced cohort than in the full cohort (estimate = 0.92, 95% CI, 0.21–1.64; *P* = .01).

For the Social Support Inventory, men had slightly lower scores on average compared with women (SSI male effect = −0.09), but Social Support Inventory scores among men increased over time at a higher rate than among women. Social Support Inventory scores were typically 0.45 (95% CI, −0.79 to 1.71) units higher among men than women for each 3-month follow-up period (*P* = .48). In the reduced cohort, this effect was a 0.65-unit increase (95% CI, −1.06 to 2.35; *P* = .46). In the full cohort, quality of life scores were typically 0.46 (95% CI, −1.16 to 2.11; *P* = .58) units higher among men than among women for each 3-month follow-up period. In the reduced cohort, this effect was a 1.67-unit increase (95% CI, −0.67 to 4.04; *P* = .17). Because only 27 of the 189 LINKS participants were men, the study was not properly powered to investigate sex-specific effects.

## Discussion

After regular contact from both a CHW in a clinical and a community-based setting during 6 months in the LINKS intervention, scores for social support, hopefulness, and quality of life measures improved among participants. Follow-up measures were significantly different from baseline measures, with a standardized change of approximately 0.28 per 3 months of intervention in each of the 3 outcomes. This change represents a decrease of more than half an SD (0.56) during the 6 months of follow-up.

Improvements in emotional well-being indicators were greatest when baseline scores were low. The trend in hopefulness and quality of life during follow-up decreased with increasing age, indicating that the intervention may have a stronger initial impact on younger participants. Additionally, the men who participated in LINKS had higher scores than women on the Social Support Inventory and quality of life scales during follow-up. LINKS may affect perceived social support and quality of life differently among men compared with women. Although this effect modification was reasonably large for both the Social Support Inventory and quality of life among men, it was not significant. Only 27 LINKS participants were men; thus, we estimated an interaction effect in a small sample. Further investigation into the effect of CHW-led community–clinical linkage interventions with men is warranted. The CHWs who collected the data indicated that participants often rated their emotional well-being in the midlevel range, such as “good.” After completing the survey instrument and after the CHW established trust, participants frequently discussed serious challenges in their lives with the CHW. If these challenges had been reflected in the baseline survey, baseline scores would have been lower than reported.

Although LINKS is focused on emotional well-being rather than a diagnosed mental health issue, our results parallel the results of previous research on the effect of CHW interventions on mental health outcomes. Kangovi et al concluded that a flexible CHW intervention aimed at participant-identified social-determinant-of-health goals improved participant self-rated mental health (23). Myers et al found that a CHW mental health counselling intervention was highly acceptable to participants with a diagnosed chronic disease and hazardous/harmful drinking or probable depression (24). In a systematic review of CHWs working in mental health interventions in the US, Weaver and Lapidos found evidence suggesting that CHWs may contribute to professional teams addressing mental health issues. The authors highlighted that CHWs serving in mental health roles were acceptable to clients as demonstrated by low attrition and high rates of intervention attendance (25).

Moving forward, we encourage researchers to expand on the LINKS model in the following ways. Racist nativism should be considered as a factor that may influence the emotional well-being of Latino/a participants. Racist nativism is the “the assigning of values to real or imagined differences, in order to justify the superiority of the native, who is to be perceived white, over that of the non-native, who is perceived to be People and Immigrants of Color, and thereby defend the right of whites, or the natives, to dominance” (26). Although we did not directly measure it in LINKS, some LINKS participants whose primary language is Spanish said they struggled to get the resources they needed in English-only environments. This problem reflects anti-immigrant state policies such as the voter-approved proposition that made English the official state language in Arizona, thus requiring government activities to be in English. Laws such as this restrict immigrants’ access to social services (27).

Despite the disparities that result from racist nativism, Latino/a individuals still have a higher life expectancy than the overall US population (28). It should not be assumed, however, that this advantage will persist indefinitely or that the life expectancy of Latino/a populations would not be higher in a more equitable society. The time is now to create and translate evidence-based, emotional well-being interventions that will benefit the Latino/a community. In doing so, Latino/a protective factors, described in the Sociocultural Resilience Model (29), should be explored as an assets-based approach to health promotion with CHWs.

This study has several limitations. We did not include randomization to a comparison condition in LINKS; the potential for selection bias should be addressed in future studies. Additionally, although we chose to examine emotional well-being as a broad construct that includes sociocultural assets, this construct is not standardized, and, thus, a comparison of our results and other research is challenging. Our study also has several strengths. Our protocol included 2 follow-up surveys to examine trends over time, strengthening our research design. Our analytical models also efficiently used all available follow-up data, thereby minimizing the effect of missing data. Another strength was our low attrition rate for a community-based intervention (22% at 3-month follow-up and 9% at 6-month follow-up). Finally, an additional strength and limitation is the functionalist translation approach used for adapting all survey instruments. Although this community-responsive approach centers research on the meaning and perspectives of the participants’ native language and local dialect, using this method does preclude the use of national norms in scoring or comparisons — such as with quality-of-life instruments like the SF-8.

We found that CHW-led community–clinical linkages can result in positive emotional well-being outcomes for participants, especially for men and participants with low baseline scores. We encourage public health practitioners to consider CHW-led community–clinical linkage interventions as a potential solution to address disparities in emotional well-being. Additionally, because CHW-led community–clinical linkage interventions may be needed as a result of racist nativism, we encourage future researchers to include an examination of the effects of racist nativism and Latino/a health advantages in their study design.
